# Multislice computed tomography is useful for evaluating partial anomalous pulmonary venous connection

**DOI:** 10.1186/1749-8090-5-40

**Published:** 2010-05-18

**Authors:** Hirofumi Kasahara, Ryo Aeba, Yutaka Tanami, Ryohei Yozu

**Affiliations:** 1 Division of Cardiovascular Surgery, Keio University, Tokyo, Japan; 2 Department of Radiology, Keio University, Tokyo, Japan

## Abstract

Volume-rendered images, derived from multidetector-row computed tomography (MDCT), can facilitate assessment of the morphology of partial anomalous pulmonary venous connection and are thus useful in pre-operative planning to prevent surgical morbidity and assist post-operative evaluations.

## Introduction

Partial anomalous pulmonary venous connection (PAPVC) is usually diagnosed by echocardiography, and catheter-based angiographies are often performed for confirmation. However, echocardiography occasionally provides insufficient information due to its small field of view, insufficient resolution to identify individual pulmonary veins, and the difficulty of confirming its penetration into the connection site of the systemic venous system, especially around the hilar [1,2]. Conventional angiography remains the standard diagnostic tool, despite its inherent risks and occasional insufficient resolution for detecting faint images of the pulmonary vein in the late phase [2,3]. Post-operative evaluations of reconstructed vessels are often problematic in certain diseases. High-slice multidetector-row computed tomography (MDCT) angiography can provide more precise morphologic delineation due to its non-invasive nature and high spatial and temporal resolution. We herein report our current experience of applying MDCT angiography for pre- and post-operative evaluations in patients with PAPVC.

## Case Reports

**Figure 1:**
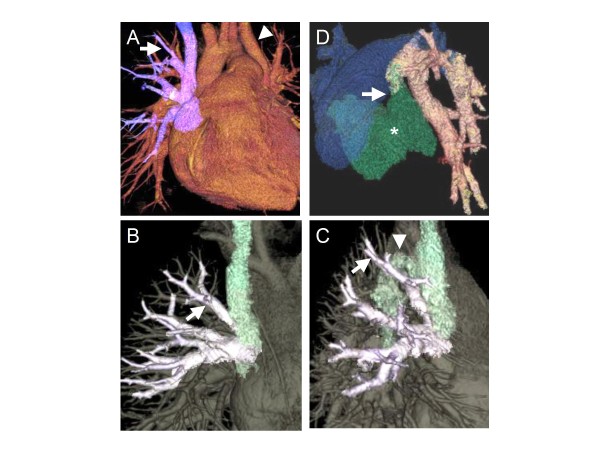
**Volume-rendered image multidetector-row computed tomography (MDCT). **   A - Blue-colored vessels return to the superior vena cava (SVC). The arrow indicates a high connected pulmonary vein. The arrowhead indicates the left persistent SVC.  B, C - Target vessel-enhanced image derived from MDCT. The arrow indicates the highest SVC site connection, which without this image is at risk of being mistaken for the azygos vein. The arrowhead indicates the azygos vein. D - A target component-enhanced image derived from MDCT. The arrow indicates the anastomosis site of the left atrium appendage with the vertical vein. The asterisk (green) indicates the left atrium.

A 10-year-old girl was diagnosed with a sinus venous septal defect with PAPVC of the right upper lobe vein to the superior vena cava (SVC). Echocardiography and catheter angiography demonstrated a large defect with a significant left-to-right shunt. A pre-operative MDCT angiogram revealed a higher SVC site connection (Fig. 1A), not detected in other examinations, and this child had a persistent left SVC; hence the decision to avoid the complex technique of patch placement within the relatively small right-side SVC. During the operation, the SVC was dissected extensively to beyond the high connected pulmonary vein under the guidance of the MDCT image. Caval division and atriocaval anastomosis described by Warden [4] was performed according to pre-operative planning.

A 4-year-old girl had anomalous drainage of the right upper pulmonary vein to the SVC, underwent a repair operation. During the operation, a pair of right upper pulmonary veins draining into the proximal site of the SVC was found following extensive dissection around the atriocaval junction, but a pre-operative MDCT angiogram indicated an additional higher SVC site connection (Fig. 1B). The higher one was erroneously considered to be the azygos vein without MDCT angiography due to its high connection onto the posterior aspect of the SVC in operative findings. The connection site of the azygos vein was found to be slightly distal following additional dissection around the SVC (Fig. 1C).

A 28 year-old woman had anomalous drainage of the left upper pulmonary vein as a vertical vein into the brachiocephalic vein, and underwent surgical repair. Post-operative enhanced images of the target vessels, derived from MDCT, demonstrated the patent pulmonary vein anastomosed to the left atrial appendage (Fig. 1D), which had been missed on echocardiography.

## Discussion

Having consecutively applied MDCT angiography to 12 patients with PAPVC, we note that the high quality of the images facilitates precise pre-operative planning, making MDCT useful for evaluating surgical morbidity post-operatively with only minor failures.

Pre-operative information obtainable via MDCT angiography concerning the number and sizes of pulmonary veins into the SVC, the precise connection sites, and the spatial relations between the PAPVC, azygos vein and cavoatrial junction, is useful for surgical planning and preventing surgical complications, as well as diagnosing various types of PAPVC. Our experience suggests there is a possibility of missing reconstruction of higher connected pulmonary veins without sufficient preoperative information because surgeons are likely not to extend dissection around the SVC because it is associated with a certain risk of injury to the phrenic nerve unless adequate preoperative information is available. To prevent cavoatrial stenosis in the Warden procedure, cavoatrial anastomosis must be accomplished without tension, requiring extensive dissection around the SVC toward the brachiocephalic vein and the jugulo-subclavian junction [5]. The three-dimensional images from MDCT angiography clearly reveal the morphology of pulmonary venous connection without any need for mental reconstruction, facilitating precise planning of the operation.

MDCT angiography is useful for post-operative evaluations to clearly demonstrate patency or occlusion of the connected pulmonary veins and SVC. No symptoms are likely to emerge in a patient with bilateral SVC, such as the present case (a 10-year-old girl), even with the reconstructed vena cava totally occluded or complicated by severe stenosis [5]. Occlusion of the reconstructed pulmonary vein is also likely to be symptom-free, if its drainage area is limited. Post-operative transthoracic echocardiography occasionally provides a poor image, especially when using intracardiac patch baffling, making it impossible to rule out significant surgical morbidity. Catheter angiography may reveal reconstructed vessels but is relatively invasive soon after the operation especially for small patients

Advanced magnetic resonance (MR) angiography devices have potential as new standard tools for visualizing pulmonary vein pathologies, since they allow non-invasive assessment of anatomy and pathophysiology at the same time [6]. When it comes to surgical planning, however, surgeons are likely to prefer high resolution MDCT angiography, since it offers a superior spatial resolution with clear morphology [2,7]. Although there are concerns regarding the higher degree of patient exposure to radiation than with MRI, its faster scanning and reduced sedation requirements are beneficial, especially in ill, uncooperative or small patients [7], and its wider availability is also an advantage.

## Conclusion

MDCT angiograms that facilitate improved assessment of the morphology of PAPVC are useful in pre-operative planning to prevent surgical complications, and assist post-operative evaluations.

## Consent

Written informed consent was obtained from the patient for publication of this case-report and any accompanying images. A copy of the written consent is available for review by the Editor-in-Chief of this journal

## Competing interests

The authors declare that they have no competing interests.

## Authors' contributions

All authors have read and approved the final manuscript. HK performed the operation and has been involved in drafting the manuscript. RA performed the operation, has been involved in drafting the manuscript. YT performed analysis of CT scan images, and has been involved in drafting the manuscript. RY performed the operation, and has given the final approval to publish the manuscript.
